# Histone Deacetylase Inhibitors Ameliorate Morphological Defects and Hypoexcitability of iPSC-Neurons from Rubinstein-Taybi Patients

**DOI:** 10.3390/ijms22115777

**Published:** 2021-05-28

**Authors:** Valentina Alari, Paolo Scalmani, Paola Francesca Ajmone, Sara Perego, Sabrina Avignone, Ilaria Catusi, Paola Adele Lonati, Maria Orietta Borghi, Palma Finelli, Benedetta Terragni, Massimo Mantegazza, Silvia Russo, Lidia Larizza

**Affiliations:** 1 Research Laboratory of Medical Cytogenetics and Molecular Genetics, IRCCS Istituto Auxologico Italiano, Via Ariosto 13, 20145 Milan, Italy; v.alari@auxologico.it (V.A.); sara.perego92@gmail.com (S.P.); i.catusi@auxologico.it (I.C.); or palma.finelli@unimi.it (P.F.); 2 Department of Neurophysiology and Diagnostic Epileptology, IRCCS Foundation C. Besta Neurological Institute, 20133 Milan, Italy; Paolo.Scalmani@istituto-besta.it (P.S.); Benedetta.Terragni@istituto-besta.it (B.T.); mantegazza@ipmc.cnrs.fr (M.M.); 3 Child and Adolescent Neuropsychiatric Service (UONPIA), Fondazione IRCCS Ca’ Granda Ospedale Maggiore Policlinico, Via Pace 9, 20122 Milan, Italy; p.ajmone@policlinico.mi.it; 4 Department of Neuroradiology, Fondazione IRCCS Ca’ Granda Ospedale Maggiore Policlinico, 20122 Milan, Italy; sabrina.avignone@policlinico.mi.it; 5 Department of Clinical Sciences and Community Health, University of Milan, 20122 Milan, Italy; paola.lonati@libero.it (P.A.L.); o.borghi@auxologico.it (M.O.B.); 6 Immunorheumatology Research Laboratory, IRCCS Istituto Auxologico Italiano, Via Ariosto 13, 20145 Milan, Italy; 7 Department of Medical Biotechnology and Translational Medicine, University of Milan, 20133 Milan, Italy; 8 Institute of Molecular and Cellular Pharmacology (IPMC), CNRS UMR7275, Inserm, LabEx ICST, Université Cote d’Azur (UCA), Sophia Antipolis, 06560 Valbonne, France

**Keywords:** Rubinstein-Taybi, intellectual disability, iPSC-neurons, histone deacetylase inhibitors, trichostatin A, valproic acid, morphological abnormalities, hypoexcitability, partial rescue

## Abstract

Rubinstein-Taybi syndrome (RSTS) is a rare neurodevelopmental disorder caused by mutations in *CREBBP* or *EP300* genes encoding CBP/p300 lysine acetyltransferases. We investigated the efficacy of the histone deacetylase inhibitor (HDACi) Trichostatin A (TSA) in ameliorating morphological abnormalities of iPSC-derived young neurons from P149 and P34 *CREBBP*-mutated patients and hypoexcitability of mature neurons from P149. Neural progenitors from both patients’ iPSC lines were cultured one week with TSA 20 nM and, only P149, for 6 weeks with TSA 0.2 nM, in parallel to neural progenitors from controls. Immunofluorescence of MAP2/TUJ1 positive cells using the Skeletonize Image J plugin evidenced that TSA partially rescued reduced nuclear area, and decreased branch length and abnormal end points number of both 45 days patients’ neurons, but did not influence the diminished percentage of their neurons with respect to controls. Patch clamp recordings of TSA-treated post-mitotic P149 neurons showed complete/partial rescue of sodium/potassium currents and significant enhancement of neuron excitability compared to untreated replicas. Correction of abnormalities of P149 young neurons was also affected by valproic acid 1 mM for 72 h, with some variation, with respect to TSA, on the morphological parameter. These findings hold promise for development of an epigenetic therapy to attenuate RSTS patients cognitive impairment.

## 1. Introduction

Rubinstein-Taybi syndrome (RSTS1 MIM #180849, RSTS2 MIM #613684) is a multisystem developmental disorder characterized by moderate to severe intellectual disability (ID), facial dysmorphism, small stature, skeletal dysplasia, multi-organ malformation, and cancer predisposition [1]. It affects 1:125.000 individuals, but the prevalence in institutionalized individuals rises to 1:300 [2]. RSTS is caused by monoallelic pathogenic variants in either *CREBBP* (RSTS1) or *EP300* (RSTS2) genes [3,4], which encode the paralog CBP and p300 chromatin modifiers acting as transcriptional coactivators and histone and non-histone lysine (K) acetyltransferases (HATs or KATs) [5]. Both RSTS genes are highly dosage-sensitive [6], highlighting a loss-of-function mutation mechanism. RSTS1, the main entity, accounting for 60% of the clinically diagnosed patients [7], presents an overall phenotype more severe than RSTS2, which is molecularly confirmed in up to 10% of the cases [8]. Despite the pronounced RSTS clinical expressivity, mainly dependent on locus and allele heterogeneity, out of the multiple clinical signs, cognitive impairment is shared by both RSTS1 and RSTS2 and displayed by most *CREBBP*-mutated patients. RSTS belongs to the increasingly expanding group of disorders of the epigenetic machinery, in the majority of which co-occurrence of intellectual disability (ID) is observed [9,10]. Insights into the relationship between mutated epigenetic genes and neurological dysfunction have been unveiled by the finding that a subset of these genes, enriched in those highly intolerant to variation and encoding proteins with a “dual” “writer” and “reader” epigenetic function, are co-expressed in multiple tissues [11]. The emerging hypothesis is that defect in any epigenetic genes, in which expression is coordinately orchestrated, disrupts the precise balance needed for neuronal homeostasis leading to cognitive impairment, a feature shared by the majority of epigenetic machinery disorders [9]. The Rubinstein-Taybi *CREBBP* and *EP300* genes are highly dosage-sensitive; their proteins perform as writers and readers of acetylated K motifs by their KAT and bromo domains, respectively. Studies on conditional knock-out mice demonstrated that cbp loss leads to decreased neural histone acetylation and also revealed that CBP has an essential role for both embryonic neural differentiation and neural functions during adult life [12]. Thus, the RSTS genes are uniquely positioned to disentangle the mechanistic basis of intellectual disability. In addition, RSTS has been intensively investigated as model for potentially treatable cause of intellectual disability. As defective KATs tip the balance in favor of the counteractive histone deacetylases (HDACs), determining closed inactive chromatin at target loci, postnatal intervention with HDAC inhibitors (HDACi) directly targeting downstream epigenetic abnormalities, has been envisaged and successfully applied in different *cbp* +/− mouse models with improvement of some neurological abnormalities [13,14]. Epidrugs, such as Trichostatin A (TSA), belonging to the hydroxamates group of HDACi whose main target are HDACs class I and II [15], tested in vitro on cortical precursors of *cbp* +/− mice rescued the defective neurogenesis, confirming that the differentiation defect was due to decreased histone acetylation [16]. These seminal breakthroughs were extended to humans as in vitro TSA treatment of lymphoblastoid cell lines from *CREBBP*-mutated patients induced the reversal of the histone acetylation deficits [17].

To investigate the effect of TSA on the cells most relevant to the cognitive deficit of the patients, we took advantage of the iPSC-derived neuronal model generated from RSTS patients displaying mental impairment of variable severity [18]. RNA-Seq analysis of iPSC derived neurons (iNeurons) from a set of RSTS patients showed a defective and altered neuroprogenitor to neuron transcriptional program with respect to healthy iNeurons due to a lower number of regulated genes and a signature compounded by improperly upregulated genes involved in neural migration and axonal targeting and downregulated RNA and DNA metabolic genes [19]. Out of the modeled patients, we selected the *CREBBP*-mutated patient 149 (aka P149), who harbors exon 18 c.3474 G>A, p.(Trp1158ter) truncating mutation and manifests the most severe ID [7] to assess whether short- and long-term exposure of his iPSC-derived neural progenitors to TSA might ameliorate the morphological and electrophysiological alterations exhibited by young and mature neurons from characterized RSTS patients [18]. We report that administration of TSA 20 nM for one week to 2 independent P149 neural progenitor lines determined in both replicas a partial rescue of some morphological alterations displayed by untreated neurons, and this effect is on the whole replicated in differentiating neurons from another *CREBBP*-mutated patient. Chronic treatment by TSA 0.2 nM supplemented to the medium of P149 neural progenitors for 6 weeks yielded a beneficial effect on post-mitotic neurons, too, as indicated by significant rescue of the defective electrophysiological alterations recorded in untreated patient neurons. Sodium valproate, a short chain fatty acid group HDACi, primarily used as medication to treat epilepsy and bipolar disorder, administered at 1 mM [15] for 72 h to P149 differentiating neurons, also showed a positive effect, greater or smaller than TSA, depending on the morphological parameter. We discuss the translational relevance of these results, particularly considering that protein acetylation/deacetylation plays a critical role in neurons for synaptic plasticity and memory [20], and the cognitive impairment of RSTS patients may not be simply ascribed to defects arisen during development, but also to continuous requirement of appropriate KAT levels in the postnatal life [13,21].

## 2. Results

### 2.1. Patients’ Molecular Characterization and Overall Clinical Phenotype

The clinical and molecular characterization of patient 149, carrying *CREBBP* exon 18 nonsense mutation c.3474G > A, p.(Trp1158ter) predicting a CBP protein lacking bromo, KAT, and downstream domains, has been reported (Reference [7], Supplementary Table S1a–d), as well as that of patient 34, carrying *CREBBP* exon 27 nonsense mutation c.4435G > T, p.(Gly1479ter) predicting a CBP protein lacking the terminal part of KAT domain and downstream domains [22]. A truncated protein was not detected by Western blot on P149 and P34 iPS cell extracts, but densitometric analysis assessed a 68% and 46% reduced amount of full length CBP wild type protein, respectively [23].

P149, currently an 11-year-old male, was born to healthy non-consanguineous parents, who have two healthy sons, by c-section at 38 weeks due to maternal hypertension during pregnancy, brain malformations, and megacystis at fetal echography, hypoplasic corpus callosum, and cerebellar vermis hypoplasia at RMN. Apgar score was 8–9. At birth, weight was 2830 g, height 47.50 cm, and cranial circumference 33 cm. At neonatal hospitalization the presence of all distinctive RSTS craniofacial dysmorphisms, the diagnostic sign of broad thumb, broad bifid hallux, and broad distal phalanges, together with post-axial polydactyly, cryptorchidism, and other clinical findings, raised the suspicion of RSTS, which was confirmed by molecular diagnosis when the infant was one month and 20 days old. The child manifested growth delay with no movement till 5 months and was subject to PEG (Percutaneous Endoscopic Gastrostomy) till age 2 due to deglutition problems and GER (Gastro Esophageal Reflux); his speech was restricted to a few words, and he suffered from several medical complications, including nystagmus, exotropia, glaucoma, and recurrent airways infections.

P34, now a 17-year-old male, was born to healthy non-consanguineous parents, who have an elder and a younger healthy son. Growth delay was noted at 2 months due to repeated vomiting. Clinical diagnosis of RSTS, suspected by large bifid and abducted thumb, was confirmed by molecular diagnosis when the child was one year old. Global psychomotor impairment and speech delay requested physiotherapy, psychometric test, logopedia, and occupational therapy. Currently, the boy has small stature and increased weight.

### 2.2. Patients’ Neurological Assessment, EEG, and Neuroradiological Findings

Neuropsychiatric phenotype of both patients has been described in the context of a cohort of RSTS patients [24].

At the age of first neuropsychiatric assessment P149 was 3 years old and motor milestones, language, and communicative abilities were significantly delayed. He did not present seizure, EEG abnormalities, nor neurologic specific signs, except microcephaly. The developmental assessment showed a severe psychomotor delay: he obtained a GQ (General Quotient of Development) of 25 with a mental age of 9.6 months (corresponding to a severe delay according to ICD 10). His adaptive skills (tested using Vineland scale) were lower than expected in all scales, and his adaptive level corresponded to 12 months. Moreover, the behavior assessment (CBCL, SCQ) showed high levels of withdrawn behaviors and autistic spectrum disorders.

**Figure 1:**
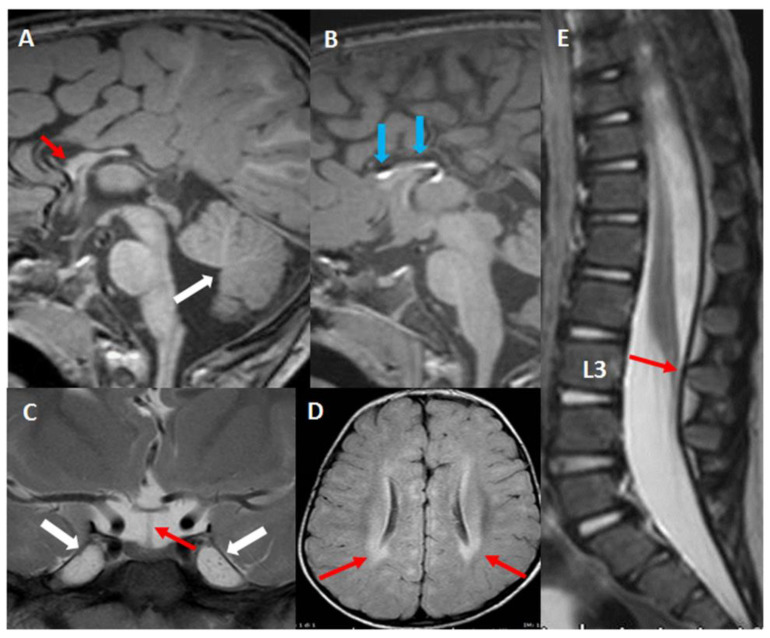
Neuroradiological findings of P149 (**A**,**B**) sagittal T1 3D TFE images showing corpus callosum partial agenesis (red arrow) and associated lipoma (blue arrows), mild cerebellar vermis extra rotation (white arrow). (**C**) T2 weighted coronal image Meckel’s cave ectasia (white arrows) and elongated pituitary stalk (red arrow). (**D**) FLAIR 2D image showing shaded and diffuse posterior white matter hyper-intensity (red arrows). (**E**) T2 weighted sagittal image showing low-lying conus medullaris ending at L3 (red arrow).

Brain MRI showed severe corpus callosum (CC) dysmorphism represented by partial agenesis and CC lipoma, mild cerebellar vermis extra rotation and hypoplasia (Figure 1A,B). Minor neuroradiological findings were moderate Meckel’s cave ectasia and minimal elongated pituitary stalk (Figure 1C). T2 and FLAIR showed shaded and diffuse posterior white matter hyperintensity (Figure 1D). Spine MRI showed low-lying conus medullaris ending at L3 (vertebral body), without terminal filum thickening (Figure 1E).

Contemporary to reprogramming patient’s blood cells to generate iPSC-derived neurons, a neuropsychiatric observation was performed. The patient was 6 years old and he still showed a significant delay: he walked without support, but the steps were unstable, expressive language was more compromised than receptive language (he produced only few words), and his behavior was characterized by attention deficits and autistic traits.

Patient 34 was 8 years old at the time of assessment. He showed a moderate ID (IQ = 46) and an adaptive level lower for his age with better abilities in communicative and socializing skills. His expressive and receptive language was delayed. No autism spectrum disorder nor specific challenging behaviors were observed. He did not present neurological signs except clumsiness and microcephaly. His sleep EEG showed spike and waves (SW) and generalized poly spike waves (PSW) and a theta delta monomorphic activity on posterior regions; no seizures were present. Neuroradiology showed corpus callosum dysmorphism, slight hypoplasia of cerebellar vermis, and spinal cord tethered at L3 vertebral body.

### 2.3. Differentiation of Patients’ iPSCs to Cortical Neurons

Reprogramming P149, P34, and healthy individuals’ blood cells to iPSC and iPSC characterization have been reported [18,23]. Details on iPSC lines P149 7 and P34 2 can be accessed on the Human Pluripotent Stem Cell Registry https://hpscreg.eu/ (accessed on 18 October 2019 under the names IAIi004-A and IAIi002-A, respectively (see Section 4.3, Materials & Methods). Differentiation of Neural Progenitor lines from patients and controls to cortical neurons according to the described protocol [18] confirmed the expression of the expected stage-specific immunofluorescence (IF) markers.

### 2.4. Short-Term and Long-Term TSA Treatments

Neural progenitors derived from initial differentiation (35 days) of iPSC colonies from patients and controls, were thawed and cultured to carry on differentiation to cortical neurons [18]. After three days of cell recovery control and patient neural progenitor cultures underwent in parallel both the short-term treatment with TSA 20 nM for 7 days till the stage of young neurons and the long-term treatment with TSA 0.2 nM for 6 weeks till to mature neurons. A replica of each TSA-treated culture had the medium supplemented with vehicle (designed as NT). The bifurcated experimental workflow leading to 45 days neurons suitable for morphological analysis and >75 post-mitotic neurons for electrophysiological analysis is depicted in Figure S1A,B. For acute treatment, we selected TSA 20 nM according to IC50 (half maximal inhibitory concentration) values around this concentration and because this dosage was shown to be effective on *cbp* +/− mouse cortical precursors cultured for 4 days [16]. Preliminarily, we also tested TSA 10 nM to verify a possible higher sensitivity of human cells, but the Trypan blue exclusion test did not evidence significant differences in the viable cell count in the CTRLs and P149 neural progenitor lines progressing to early neurons exposed to the two TSA concentrations, compared to untreated samples (Supplementary Figure S1C). Thus, since it did not impair cell survival, the higher 20 nM dosage was selected to investigate TSA effect on the morphology of young (45 days) neurons from CTRLs, P149 lines 6 and 7, and P34 line 2.

No significant differences in cell viability as compared to untreated samples were also recorded in CTRLs and P149 lines after chronic (6 weeks) treatment by supplementation of TSA 0.2 nM to neural differentiation medium (Figure S1D).

#### 2.4.1. Short-Term TSA Treatment: Effect on Neuronal Differentiation and Morphological Parameters of P149 and P34 Young Neurons

**Figure 2:**
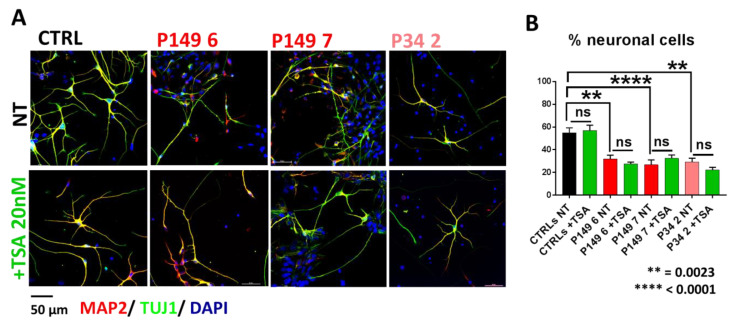
Percentage of neuronal cells in controls, P149 6 and 7, and P34 2 neural progenitor cell lines cultured for one week in neural differentiation medium without (top panels) and with (bottom panels) TSA 20 nM. (**A**) Immunofluorescence of neuronal markers MAP2 (red) and TUJ1 (green). Nuclei are counterstained with DAPI. Scale Bar 50 µm. Patients lines show, as compared to controls, a higher number of DAPI positive cells unstained by MAP2 and TUJ1 antibodies (top panels) persisting after TSA treatment (bottom panels). (**B**) Graphs showing a highly significant (**** *p* < 0.0001) and significant (** *p* = 0.0023) decrease in cells identified as neurons by MAP2/TUJ1 immunostaining in P149 7, and P149 6 and P34 2, respectively, as compared to controls. TSA treatment does not impact the percentage of neuronal cells in both patients and control lines (ns.: not significant) (Kruskal–Wallis non-parametric test).

Immunofluorescence (IF) analysis showed that differentiating neurons from both P149 lines 6 and 7 and P34 line 2 co-express, like control neurons, the pan neuronal markers TUJ1 and MAP2, which indicates that these cells have neuronal features (Figure 2A). However, we observed a sharp reduction in the percentage of neuronal cells in P149 lines 6 and 7, and in the P34 2 line, because, compared to CTRLs, there were more DAPI-positive cells unstained by the TUJ1 and MAP2 antibodies. In fact, the quantification displayed in Figure 2B shows that the percentages of TUJ1/MAP2 stained cells on the total cells counted in acquired field images equalized across samples (*n* = 30) are highly significantly reduced in P149 7 and significantly reduced in P149 6 and P34 2 compared to CTRLs (*p* value < 0.0001, and *p* value = 0.0023, respectively). The Kruskal–Wallis non-parametric test was used for multiple comparisons (patients versus controls and TSA-treated versus untreated samples), referring to morphological markers. Exposure of controls and patients lines to TSA 20 nM for one week did not yield any beneficial effect on neural differentiation of patient lines nor controls.

To evaluate the effect of TSA on the morphology of neural progenitors after 7 days of culture, confocal microscopy images of MAP2/TUJ1-positive 45 days neurons from coverslips of patient and control samples were analyzed using the Image J plugin Analyze Skeleton [25] (Figure S2).

Besides the morphological parameters we have previously identified as biomarkers of young iNeurons from RSTS patients, namely reduced branch length and increased branch number [18], we included evaluation of the nucleus size, as reduced nuclear area has been considered a marker of differentiating neurons from other epigenetic syndromes, such as Rett syndrome [26].

**Figure 3:**
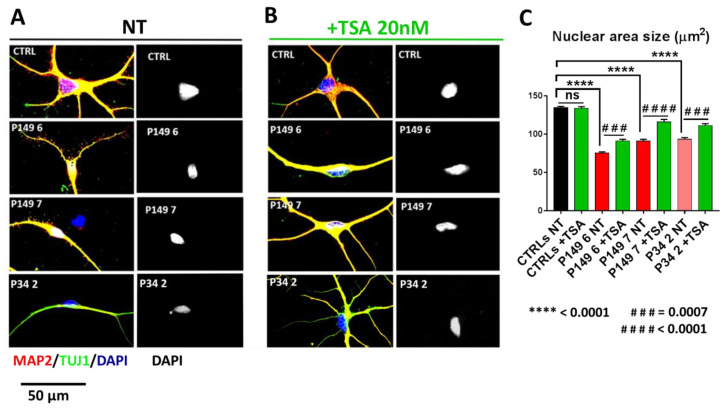
Partial rescue of the reduced nuclear size of 45 days neurons from P149 neural progenitor cell lines 6 and 7 and P34 line 2 by one week treatment with TSA 20 nM. Immunofluorescence of MAP2 (red) and TUJ1 (green) markers in (**A**) untreated (NT) controls and P149 and P34 lines; (**B**) TSA-treated controls and P149 and P34 lines. Nuclei are counterstained with DAPI. Scale Bar 50 µm. (**C**) Nuclei analysis, performed on blue single channel binarized images, using the “Analyzed Particles” function, scoring at least 50 neurons per sample, shows highly significant decrease in nuclear area size (µm^2^) in all the *CREBBP*-mutated cell lines with respect to control lines. Effect of genotype: **** *p* < 0.0001. All TSA-treated patients’ lines show significant increase in nuclear size as compared to untreated replicas. Effect of TSA treatment on *CREBBP* +/− lines: ### *p* = 0.0007, #### *p* < 0.0001. No effect on CTRLs (ns = not significant) (Kruskal–Wallis non-parametric test).

As displayed by Figure 3A, neurons from both P149 lines and P34 2 show as compared to CTLRs neurons a visible reduction of nuclear area, quantified by scoring 50 neurons per sample, as highly significant (*p* value < 0.0001) (Figure 3C). TSA treatment partially rescued the abnormal nuclear size in all patients lines (Figure 3B). The significant differences between untreated and treated patients samples are indicated in Figure 3C. In detail, a significant increase in TSA-treated P149 6 (from 75 to 91 µm^2^) and P34 2 (from 93 to 111 µm^2^) lines and a highly significant increase in TSA-treated P149 7 line (from 91 to 116 µm^2^) was computed (Figure 3C).

**Figure 4:**
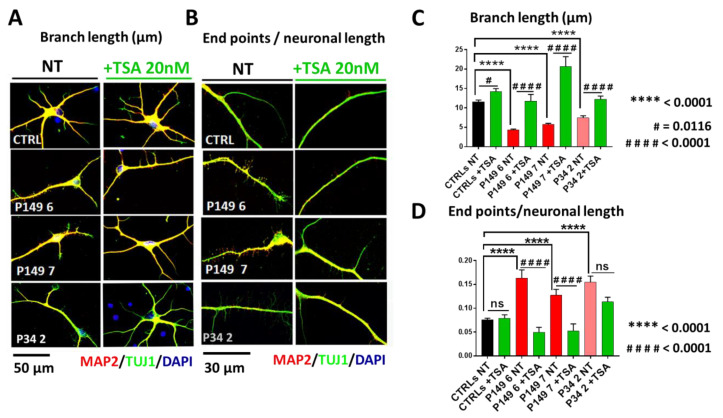
Aberrant branch length and end point number/neuronal length in *CREBBP*-mutated patients with respect to controls lines and partial rescue after TSA treatment. (**A**–**C**) Immunofluorescence of MAP2 (red), TUJ1 (green), and DAPI (blue)—scale bar 50 µm—shows decreased branch length in all patients lines with respect to controls, quantified as highly significant by Kruskal–Wallis test. Effect of genotype is represented by **** *p* < 0.0001. TSA treatment determines a significant correction of this morphological aberration. Effect of TSA treatment is represented by # *p* = 0.0116, #### *p* < 0.0001. (**B**–**D**) Increased number of end points/neuronal length in all patients lines with respect to controls lines is apparent at IF—scale bar 30 µm—and results highly significant at Kruskal–Wallis test. Effect of genotype: **** *p* < 0.0001. (**D**) The graphs also quantify the significant reduction in the end points/neuronal length ratio in both P149 lines and a trend to correction in P34 2 after TSA treatment. Effect of TSA treatment: ns = not significant, #### *p* < 0.0001.

Other morphological parameters, i.e., the branch length and the number of end points with respect to the neuronal length, computed by scoring about 50 neurons per sample, are shown in Figure 4 A,B for untreated and TSA-treated CTRLs, P149, and P34 neurons. Respect to controls the length of branches was significantly diminished in all patient neurons (*p* value < 0.0001) and significantly increased (*p* value < 0.0001) upon TSA treatment (Figure 4C). TSA enhanced branch length also in controls neurons. The ratio between end points number and neuronal length was significantly increased in both 149 lines and P34 2 line (*p* value < 0.0001) with respect to controls. TSA significantly reduced this parameter for P149 lines (*p* value < 0.0001), while P34 neurons showed a trend to correction of this ratio, as quantified by the graphs in Figure 4D. The total neuronal length does not change between CTRLs and P149 neurons, while P34 neurons are smaller and become significantly longer after TSA treatment (Supplementary Figure S3).

As regards the layout or number of dendrites growing out from the cell body, we noticed that control neurons at this stage of neural differentiation mainly comprise multipolar neurons, while both P149 lines (more 6 than 7) are enriched in unbranched or uni-polar/bipolar neurons. Instead, P34 2 has a higher percentage of multipolar cells, more similarly to controls (Supplementary Figure S4A,B). This morphological parameter, referring to the elaboration of the dendritic tree, does not appear to be influenced by TSA treatment in patients, as well as in control neurons.

#### 2.4.2. Short-Term VPA Treatment: Effect on Morphological Parameters of P149 Young Neurons

**Figure 5:**
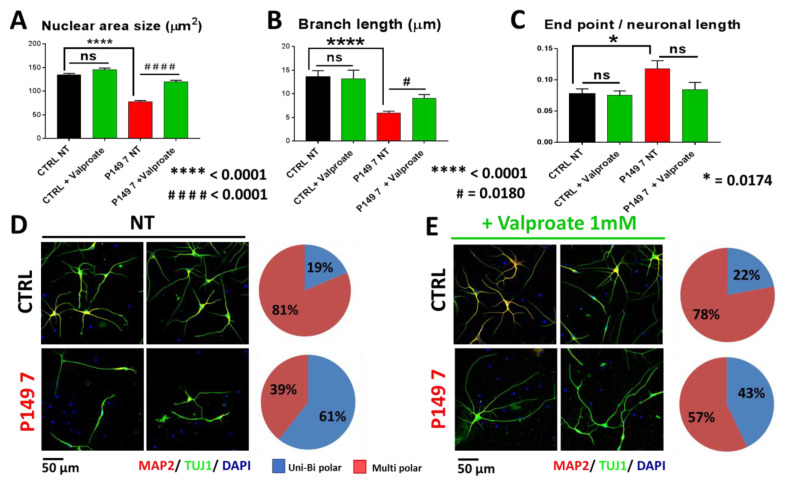
Valproate 1 mM effect on different morphological parameters of 45 days neurons from CTRL and P149 line 7 after 72 h treatment. The graphs quantify in patient’s neurons a highly significant decrease in (**A**) nuclear area size and in (**B**) branch length with respect to control (genotype effect: **** *p* < 0.0001) and a slight increase in (**C**) the ratio end point/neuronal length (* *p* = 0.0174). VPA treatment on P149 7 line significantly increases (**A**) the nuclear area size (#### *p* < 0.0001) and (**B**) the branch length (# *p* = 0.0180), while it does not yield a significantly correction of (**C**) the number of end points with respect to the neuronal length (ns = not significant) (**D**,**E**) amelioration of neuron layout with multipolar neurons increasing from 39% (NT) to 57% (VPA-treated). Immunofluorescence of MAP2 (red) and TUJ1 (green) markers, DAPI counterstaining. Scale bar 50 µm.

In order to check whether amelioration of morphological alterations of P149 neurons by TSA might be promoted by other HDACi at a similar or different extent, valproic acid (VPA), which IC50 is in the 0.5–2 mM range, was administered at 1 mM [15] to neural progenitors from control and P149 line 7 for 72 h. VPA-treated neurons showed a highly significant increase in nuclear area (*p* value < 0.0001) (Figure 5A) and a significant increase in branch length (*p* value = 0.0180) (Figure 5B). Conversely, no correction of the end points with respect to neuronal length was apparent (Figure 5C). Moreover VPA exposure significantly improved the layout of P149 line 7 neurons, with an increase in multipolar neurons, which are also slightly enhanced in control neurons, from 39% to 57% (Figure 5D,E). The significant differences in the percentage of multipolar and uni-bipolar neurons between controls and P149 7 line (genotype effect) and between untreated and VPA-treated lines (treatment effect) are shown in Supplementary Figure S4C,D.

#### 2.4.3. Chronic Long-Term TSA Treatment: Effect on the Electrophysiological Features of P149 Post-Mitotic Neurons

Post-mitotic (>75 days) neurons from both P149 neural progenitor cell lines displayed the expected pattern of expression of the cortical and the presynaptic markers CUX-1 and SYP; most cells showed positive immunostaining for the excitatory marker v-GLUT and a few for the inhibitory marker GAD65/67. At prolonged culture times, glial cells are evidenced by positive immunostaining for the GFAP astrocytic marker (Figure S5). All these stage-specific markers of differentiated neurons can be detected by IF in post-mitotic P149 neuronal cultures, likewise observed for all other modeled RSTS patients [18].

The percentage of MAP2 positive cells in both P149 post-mitotic neurons could not be assessed, as for young neurons by IF analysis, due to the intricate network with clusters of overlapping cells at this stage. Thus, IF was replaced by cytofluorimetric analysis using MAP2 antibody, which evidenced 49.88% positive cells in CTRLs, while this percentage dropped to 32.91% and 35.9% in P149 6 and 7 neurons, respectively, indicating a defective neural differentiation process.

**Figure 6:**
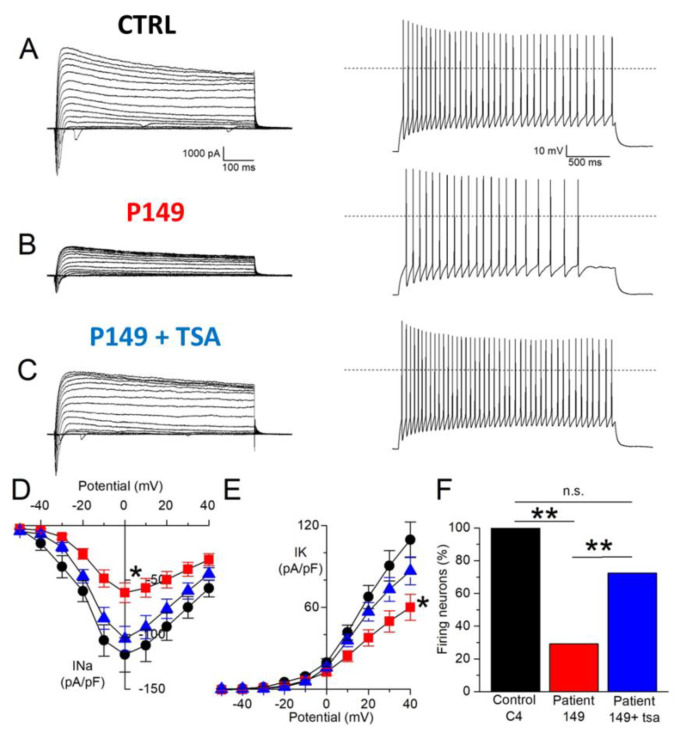
Electrophysiological characterization of neurons from control, P149, and P149 + TSA after 75 days of differentiation. (**A**) Left: representative total whole-cell ionic currents elicited in a control neuron with depolarizing voltage steps between−70 and +60 mV, from a holding potential of −70 mV; right, representative discharges of action potentials (APs) evoked with current-clamp recordings in a control neuron by the injection of a 2.5 s-long 50 pA depolarizing current step. (**B**) Similar representative recordings in a neuron from patient 149. (**C**) Similar representative recordings in a neuron from P149 + TSA. (**D**) I–V plot displaying the quantification of sodium current densities at different potentials for control (black circles), P149 (red squares), P149 + TSA (blue triangles): maximal current density for control C (118.4 ± 15.9 pA/pF, *n* = 12), P149 (61.4 ± 9.0 pA/pF, *n* = 24), and P149 + TSA (103.7 ± 11.4 pA/pF, *n* = 29); C vs. #149 *p* = 0.01, C vs. #149 + TSA *p* = 0.68, P149 vs. P149 + TSA *p* = 0.02 (ANOVA with Scheffe post-hoc test). (**E**) I–V plot displaying the quantification of potassium current densities at different potentials: maximal current density for control C (109.6 ± 13.0 pA/pF, *n* = 18), P149 (60.1 ± 9.5 pA/pF, *n* = 24), and P149 + TSAQ (86.8 ± 10.2 pA/pF, *n* = 30); C vs. P149 *p* = 0.01, C vs. P149 + TSA *p* = 0.35, P149 vs. P149 + TSA *p* = 0.19 (ANOVA with Scheffe post-hoc test). (**F**) quantification of the percentage of neurons able to generate at least one action potential for control C (13/13), P149 (7/24), P149 + TSA (21/29); C vs. P149 *p* < 0.001; C vs. P149 + TSA *p* = 0.25, P149 vs. P149 + TSA *p* = 0.007 (Fisher’s exact test with Bonferroni correction for multiple comparisons). * *p* < 0.05, ** *p* < 0.01, ns = not significant.

Neurons obtained from CTRLs, P149 6 and 7 NPC lines, all cultured with and without TSA (0.2 nM) for 6 weeks were analyzed by patch-clamp recordings to test their maturity and characterize their cellular excitability. Data from P149 6 and 7 was pooled together for comparison to CTRLs neurons. Voltage-clamp experiments showed the expression of voltage-gated sodium and potassium currents, similarly to mature neurons, as shown by the representative current traces (Figure 6A–C, left panel). Current-voltage plot for sodium current (Figure 6D) and potassium current (Figure 6E) showed reduced current amplitude for both currents in P149 compared to the control (the reduction on average was 48% for maximal sodium current and 45% for the potassium current at +40 mV). The treatment with TSA almost completely rescued the sodium current at the control values, and there was a trend towards the rescue of potassium currents.

Current-clamp experiments confirmed the maturity of differentiated control neurons because all those recorded were able to generate at least one action potential in response to injection of depolarizing current steps (Figure 6A, right panel, and Figure 6F). A lower percentage of neurons from P149 produced action potentials compared to control neurons, showing that CBP reduction caused by early truncating mutation [22] impairs excitability (Figure 6B, right panel, and Figure 6F). Interestingly, the treatment with TSA rescued the excitability of the neurons from P149 (Figure 6C, right panel, and Figure 6F). Thus, the mutation impairs the expression of voltage-gated sodium and potassium currents, which are typical of mature neurons, and reduces excitability. The treatment with TSA is effective in partially rescuing these cellular dysfunctions.

## 3. Discussion

It is increasingly recognized that mammalian neurogenesis, both during embryonic development and adult life, is driven by transient reversible epigenetic modifications [27]. Acetylation and deacetylation through the antagonistic acetyltransferases (KATs), which are recruited to enhancers priming transcriptional activation, and histone deacetylases (HDACs) which promote chromatin condensation and transcriptional repression, are such dynamic modifications regulating gene expression through modification of histone marks [28]. Acetylation deficits, tipping the balance between KATs and HDACs in favor of HDACs, are a recognized cause of impaired cognition in neurodevelopmental disorders of the epigenetic machinery, like Rubinstein-Taybi [9], as well as in neurodegenerative disorders where CBP-dependent transcription is altered [21]. Thus, modifying the gene expression profiles through inhibition of HDACs has emerged as a powerful approach to therapeutically correct the altered balance. Furthermore, many HDACi, both natural and synthetic small molecules, are active in protection and regeneration of neurons [29], thus providing also a basic research tool to study the neurogenesis process.

In the current in vitro study, we examined the efficacy of a natural HDACi, TSA, to ameliorate the impaired neural differentiation consequent to CBP haploinsufficiency of two independent iPSC-derived neural progenitor cell lines from *CREBBP*-mutated patient 149, who is positioned at the extreme end of ID spectrum and also presents ASD disorder [7,24]. We also tested the effect of TSA short-term treatment on the neural progenitors from another patient (P34), harboring a nonsense *CREBBP* mutation predicting CBP haploinsufficiency, as well, but presenting with moderate ID [23,24]. We employed TSA because it is a broad spectrum HDACi, effective at low concentrations and can be easily obtained from commercial sources. Moreover, TSA was shown to rescue the impaired differentiation to cortical neurons of mice with *cbp* haploinsufficiency or genetic knockdown [16] and proved efficacious in restoring histone acetylation deficits of lymphoblastoid cell lines from Rubinstein-Taybi patients [17]. Based on disclosure of morphological and electrophysiological biomarkers in young and post-mitotic RSTS iNeurons [18], we examined the efficacy of short-term (acute) and long-term (chronic) TSA treatment in rescuing the morphological anomalies of 45 days and >75 days neurons from two independent P149 neuronal lines. TSA treatment 20 nM for 7 days and 0.2 nM for 6 weeks did not impact the viability of P149 neuronal cultures at the two time points, arguing against a toxic effect of the drug. However, impaired neural differentiation inferred by a decreased number of TUJ1/MAP2 positive cells at the stage of young neurons was found to persist in TSA-treated cultures from neural progenitors of patients 149 and 34 (Figure 2). Cytofluorimetric analysis confirmed the decreased percentage with respect to control of MAP2-positive cells in both P149 post-mitotic neuronal cultures, at a level similar to that recorded by IF in patient early neurons.

Notwithstanding, in the stage at which neurons are at low density in culture, we could monitor in the cells recognized by immunostaining as neurons a positive influence of TSA on some morphological anomalies. Differentiating neurons from both P149 6 and 7 and P34 2 progenitor lines display a reduced nuclear area, and this defect is partially j rescued in patients neurons by TSA treatment. Reduced size of the soma and of the nucleus has been pointed out as a typical feature of iPSC-neurons from *MECP2*-caused Rett syndrome patients [26] and is an important in vitro biomarker of *Mecp2* mutation in the mouse also, where it is rescued by IGF-1 (Insulin Growth Factor 1) treatment [30]. Most important, as attested by *Mecp2* neuronal model, this biomarker can provide also for *CREBBP* mutation a quantifiable phenotype to screen for compounds that rescue the “small neuronal phenotype” [30]. Rett syndrome is, like Rubinstein-Taybi, a disorder of the epigenetic machinery, and interconnection with RSTS has been highlighted by the findings of reduced CBP-bound protein CREB and phospho-CREB levels in *MECP2* mutant iPSCs and amelioration of the neuronal morphological alterations by pharmacological activation of CREB signaling [31]. *MECP2*- and *CREBBP*-defective iNeurons also share impaired neurite length and dendritic arborization. Indeed, we observed that young neurons from P149 6 and 7 and P34 2 lines display abnormal short branches with an increased number of thin protrusions (counted as number of end points) preluding to filopodia-like spines compared to control neurons. TSA treatment significantly increased the branch length in all patient lines and reduced the number of end points with respect to neuronal length in both P149 lines, while it did not significantly correct this parameter in P34 neurons (Figure 4D). This finding can be ascribed to patient 34-specific genetic background that could influence specific features, such as small neuron size. Even if TSA promotes a significant increase in the total size of P34 neurons (Figure S3), the effect of the treatment on the number of endpoints per unit of length remains insignificant (Figure 4D). The variable effect of the same HDACi across patients may reflect patient-specific (epi)genetic background, different strength of the mutation, and/or different in vitro neuronal differentiation despite application of the same protocol [32]. The results on the same statistically significant correction of several morphological parameters obtained on the two independent P149 neuronal lines underline the maintenance of patient uniqueness.

Our findings on branch length and dendritic arborization are consistent with the observations on cultured neurons from an inducible *Cbp* knock-out mouse strain, which, displayed a shorter and less complex dendritic tree than control neurons [33]. Interestingly, the impaired outgrowth and immature spines of the mouse neurons could be ameliorated by increasing the concentration in the culture medium of neurotrophic factors, making the authors conclude that the *Cbp*-deficient neurons have an impaired response to neurotrophic factors rather than an intrinsic growth defect [33]. Similarly, TSA, which, like other second generation HDAC inhibitors, promotes neural differentiation and enhances neuronal functions [15,34,35,36], significantly improved the branch length, and led to a notable reduction of the end points number with respect to neuronal length of P149 differentiating neurons, despite CBP deficiency.

No effect was exerted by TSA on the layout of differentiating neurons from both P149 NPC lines, which maintained an inverted ratio of multipolar to uni-bipolar neurons with respect to control neurons. One possible explanation for the lack of effect of TSA on neurite outgrowth, one of the critical steps in neuronal differentiation [36], keeps into account that reduced amount of CBP, the coactivator of CREB (cAMP response element protein) results in impaired calcium-induced activity dependent dendrite morphogenesis [37]. Impaired and dysregulated neuronal differentiation was revealed by transcriptome analysis of iPSC-derived neurons from a set of RSTS patients, including P149 and P34 [19]. Hence, one may hypothesize that suppression of HDAC activity by TSA may enhance the differentiation process without correcting the timeline warded off by the genetic defect.

Another HDACi, valproic acid, exerted, like TSA, a beneficial effect on the reduced nuclear area of P149 7 neurons and showed a significant effect on their branch length, but only a trend to reduction in the number of end points with respect to the neuronal length. Interestingly, different from TSA, VPA could ameliorate the neuronal layout of differentiating neurons by increasing the fraction of multipolar versus uni-bipolar neurons.

Both TSA and VPA lack specificity: classes I and IIa HDACs are shared targets, while class IIb HDACs are unique targets of TSA [15]. It has been demonstrated that TSA inhibits HDAC-6, leading to hyperacetylation of tubulin, which is a marker for stable microtubules, while sodium butyrate, an HDACi of the same group of valproic acid, does not affect HDAC-6 activity [38]. The key role of microtubules in dendrite architecture and the diverse targets of the used HDACi account for their not fully homogeneous impact on neuron morphological parameters.

As regards the second timepoint, >75 days P149 neurons cultured for 6 weeks in the presence of TSA 0.2 nM showed a significantly enhanced maturation in electrophysiological recordings compared to untreated neurons (Figure 6), in line with results obtained on TSA-treated stem cells derived neurons differentiated in culture in a minimal serum-free medium, which developed electrophysiological properties typical of firing neurons [36]. RSTS neurons, though functional and able to fire APs, show a decreased excitability [18], similarly to neurons from Rett [26] and autism spectrum disorders patients [39]. It has been recently demonstrated that depletion of CBP and p300 in forebrain excitatory neurons of adult mice results in a severe neurological phenotype associated with reduced electric activity, allowing the authors to ascribe to KAT3 proteins the maintenance of neuronal excitatory molecular identity [40]. Thus, the beneficial effect of TSA on RSTS neurons electrophysiological features holds the promise of attenuating the synaptic dysfunction to which the low excitability may contribute.

Both morphological and electrophysiological defects of P149 neurons may result from joined nucleus and dendritic abnormalities in the context of altered cell polarity during neural differentiation [41]. Mutations in genes for cell polarity proteins have been associated with cortical malformations and diverse neurodevelopmental disorders, such as microcephaly and autism [42,43,44]. Microcephaly is a universal feature of RSTS patients and autism spectrum disorder is present in about 1/3 of RSTS patients [24]: both patients 149 and 34 are microcephalic, and P149 has been assessed as ASD since early age. He also presents agenesis of corpus callosum and hypoplasia of the cerebellar vermis, i.e., cortical malformations which have been linked to cell polarity dysfunction [45]. We know that several genes acting in cell polarity and adhesion, axonal targeting, and extension, as well as genes involved in key processes of functional neurons, are dysregulated in RSTS neurons [19], implying that defective CBP primes signaling cascades affecting both neuronal migration and synaptic formation and function. The exact pathway by which TSA altering the local chromatin structure sets in motion genes or cassettes of genes that attenuate the morphological abnormalities of RSTS P149 young neurons and partially rescue the electrophysiological features of differentiated neurons is hard to decode. Future work on the transcriptome of TSA-treated versus untreated *CREBBP*-mutated iNeurons at the two timepoints of our analysis could inform on TSA-univocal differential expressed genes and pathways.

Though we have carefully selected iPSC lines scanned by high resolution array CGH (Comparative Genome Hybridization) and have discarded those with in vitro acquired genetic alterations [18], we cannot exclude more subtle epigenetic changes, documented in human pluripotent stem cells [26,46] and the occurrence of (epi) genetic aberrations during in vitro proliferation of neural progenitors to young and to mature neurons. We are also aware of the intrinsic limitations of the in vitro patient-derived iPSC model, which include the genotype-phenotype heterogeneity across patients with different mutations compared to matched controls. Keeping into account all these limitations, our data reveals a fair concordance of the morphological parameters between the two P149 lines and between P149 and P34, which supports the strength of our results. Moreover, the defects found in iNeurons of Rubinstein-Taybi patients are similarly improved by treatment with two different HDAC inhibitors.

Though TSA has been tested in several preclinical trials for neurological diseases [15], and VPA has been administered to RSTS children in an exploratory phase 2 therapeutic trial (https://ichgcp.net/clinical-trials-registry/NCT01619644 (accessed on 1 April 2012 till 1 September 2014)), their non-specific effects due to the action on multiple HDACs (a feature applicable to most HDACi), unknown off-target effects, and potential toxicity in chronic administration are limiting factors for translation in the clinical setting. Nevertheless, the reversibility of epigenetic therapy is an advantage that encourages progressing along the route of developing novel epidrugs with more restricted/selected targets or their combinations at reduced doses [47] for therapeutic strategies with minimal adverse side effects.

## 4. Materials and Methods

### 4.1. Patients’ Neuropsychiatric Assessment

The neuropsychiatric phenotype of the patients was assessed using a specific protocol that evaluates general quotient of development (GQ), behavioral aspects, neurological and electro clinical features and neuro radiological aspects.

In order to test the GQ, Griffiths’ scale [48] was used. This scale gives a GQ in patients from 0 to 8 years old. Specifically, the GQ is composed of 6 sub-quotients, one for each area investigated (locomotor, personal-social, language, eye, and hand coordination, performance, and practical reasoning). The GQ identifies how children grow in all their developmental areas. The Vineland Adaptive Behavior Scale (VABS), in its Italian adaptation and validation [49], was used to assess adaptive behavior. The VABS is a semi-structured interview with caregivers and allows to assess global adaptive behavior skills (adaptive behavior composite) and ability on four specific domains (Communication, Daily Living Skills, Socialization, Motor Skills). To assess the presence of behavioral and emotional problems, the Child Behavior Checklist (CBCL) [50] was completed by the parents. Behavioral problems on the CBCL are divided into internalizing and externalizing domains. The scores obtained in the internalizing and externalizing problem scales are then converted into T-scores (M = 50; SD = 10). T-scores above 70 are considered in the clinical range. The CBCL is commonly used to assess behavioral characteristics.

We used the Social Communication Questionnaire: Lifetime Version (SCQ) [51] to assess autism spectrum disorders (ASD).

### 4.2. Brain and Whole Spine MRI and EEG

Patients underwent brain and whole spine magnetic resonance imaging (MRI) under sedation, which was performed on a 3T unit (Philips Achieva 3.2.3.1, Amsterdam, Netherlands). The brain MRI protocol included multiplanar T1 3D TFE, TSE T2-weighted and FLAIR images, T2 3D Drive, and Diffusion-Weighted Imaging (DWI). The whole spine MRI included sagittal T1 and T2 weighted images.

Electroclinical aspects were assessed with awake and sleep EEG recordings. The EEGs were recorded using Ag/AgCl surface electrodes (impedance < 5Ώ) placed in accordance with the 10–20 International system. The examination was recorded on split-screen video-EEG. The EMGs were recorded using surface pairs of electrodes.

### 4.3. Patients’ iPSC-Derived Neural Progenitor Lines

Patient 149 (aka P149) iPSC-derived neural progenitor cell lines 6 and 7 were cultured in parallel to control neural progenitor cell lines (NPCs) for morphological analysis of differentiating (45 days) neurons and electrophysiological characterization of mature (>75 days) neurons.

Different control lines, one adult (C1) and 3 pediatric (C4, C6, C7) were used. The diction CTRL or CTRLs indicates that either one or a pool of control lines has been used.

Patient 34 (aka P34) iPSC-derived neural progenitor cell line 2 was cultured in parallel to control neural progenitor line C7 for morphological analysis of differentiating (45 days) neurons.

P149 and P34 iPSC lines fulfill all the criteria requested by the Human Pluripotent Stem Cell Registry https://hpscreg.eu (accessed on October 18 2019), where data on iPSC line 7 (official name: IAIi004-A; alternative name: IAIi004RSTS1-149-A) and iPSC line 2 (official name IAI002-A; alternative name: IAIi002RSTS1-34-A) can be accessed.

### 4.4. Differentiation of Neural Progenitor Cell Lines (NPCs) to Cortical Neurons

NPCs from patients and controls were thawed, plated at low density (5 × 10^4^ cells in 3.8 cm^2^ well), and cultured in Neural Differentiation Medium: Neurobasal medium, 2% B27, 1% NEAA, 2 mM l-Glutamine (all reagent from Thermo Fisher Scientific, Waltham, MA, USA), plus 10 ng/mL BDNF, 10 ng/mL GDNF (both from Peprotech, London, UK), 1 µM Ascorbic Acid, and 200 µM cAMP (both from Sigma-Aldrich, St. Louis, MO, USA). Neurons were maintained in culture by half media changes twice a week until 45 days (stage of young neurons) or >75 days (stage of mature neurons).

### 4.5. Drug Treatment Conditions

TSA (Sigma-Aldrich) was diluted in DMSO at a concentration of 20 μM and added to NPCs lines cultured in Neurobasal medium from controls, P149 lines 6 and 7, and P34 line 2 at 20 nM for 1 week (short-term treatment) and 0.2 nM for 6 weeks (long-term treatment).

VPA (Sigma-Aldrich) was dissolved in double distilled water to obtain a 200 mM stock solution and used at a final concentration of 1 mM. CTRL and P149 line 7 neurons were treated on day 42 and analyzed after 72 h.

### 4.6. Cell Viability

Cell count and cell viability were determined by Trypan blue dye exclusion assay using LUNA-II^TM^ Automated Cell Counter. Cells were detached using TrypLE (Life Technologies, Waltham, MA, USA) to obtain a single cell suspension and then analyzed with a LUNA-II^TM^ Automated standard protocol. Three independent counts were made at the investigated time points.

### 4.7. Cytofluorimetric Analysis

Neurons at >75 days were harvested and gently dissociated using TrypLE (Life Technologies) to obtain a single cell suspension of 1–2 × 10^6^ cells/mL. Then, cells were fixed in 2% paraformaldehyde (PFA) for 15 min at RT and permeabilized with 0.7% Tween-20 (Sigma) for 15 min at RT. Primary antibodies (Table S1) were incubated for 1 h with 10% goat serum (Invitrogen, Waltham, MA, USA), 1% bovine serum albumin, 0.5% Tween-20 in PBS. After washing, secondary antibodies (Table S1) were incubated in PBS for 30 min, and 10000 events for each tube were acquired at a medium flow rate using a BD FACS-Lyric. Analyses were performed using BD FACSuite software (BD Biosciences, New Jersey, NJ, U.S.). Forward versus side scatter (FSC vs. SSC) gating was used to identify the cells. A single fluorochrome dot plot strategy was used to identify MAP2-positive neurons (SSC vs. MAP2-FITC), and a neuron region was drawn.

### 4.8. Immunofluorescence Staining

Cells were fixed with 4% paraformaldehyde (PFA) for 20 min at 37 °C. Primary antibodies (Table S1) were diluted in 0.2% gelatin, 0.3% Triton-X 100, 20 mM Sodium Phosphate Buffer pH 7.4, 0.45M NaCl (all from Sigma), and incubated over night at 4 °C. After washing, cells were incubated with secondary antibodies (Table S1) in the same dilution buffer for 2 h at RT. DAPI was used for nuclear staining. A Nikon Eclipse TI Confocal Microscope with a 40× and/or a 20× objective was used to analyze all the images (1024 × 1024 pixels resolution). Data of control lines were pooled for comparison to P149 6 and 7 lines and P34 2 line.

### 4.9. Morphological Analysis

Neural progenitors from controls and P149 6 and 7 lines and P34 line 2 (obtained from iPSC as described [18]), were cultured in parallel for one week without and with TSA 20 nM and VPA 1 mM for 72 h. The size of the nuclei, the branch length, and the number of end points with respect to the neuronal length and the dendritic layout were the morphological parameters analyzed on coverslips from 45 days neurons immuno-stained for the neuron-specific MAP2 cytoskeletal protein and TUJ1 class-III beta-tubulin. The Skeletonize Image J plugin [25] was used for the analysis of 30–50 cells selected for each sample. Figure S2 depicts the procedure to elaborate a binary image from a single cell which neuronal identity is confirmed by positive immunostaining of the pan neuronal markers MAP2 and TUJ1. The binary image is then skeletonized to calculate the length of branches and the number of end points with respect to the neuronal length. The binary image is also used to precisely calculated the size of the nuclear area with the “analyze particles” function. The number of primary neurites growing out from the soma is counted to categorize uni-bipolar (1–2) versus multipolar (≥3) neurons, and the layout of neuron morphology in untreated and TSA-treated control and P149 lines and VPA-treated control and P149 7 line is indicated as multipolar versus uni-polar/bipolar percentage.

### 4.10. Electrophysiological Recordings

Whole-cell patch-clamp recordings were performed on 80-day-old differentiated neurons at RT (∼25 °C) using a Multiclamp 700A amplifier and pClamp 10.5 software (MolecularDevices, Sunnyvale, CA, USA) in voltage- or current-clamp configuration, as described [18,52]. Signals were filtered at 10 kHz and sampled at 200 kHz for voltage-clamp recordings or 20 kHz for current-clamp recordings. External bath solution contained (mM): 129 NaCl, 1.25 NaH_2_PO_4_, 35 glucose, 1.8 MgSO_4_, 1.6 CaCl_2_, 3 KCl and 10 HEPES, pH 7.4 with NaOH. The internal pipette solution contained (mM): 120 K-gluconate, 15 KCl, 2 MgCl_2_, 0.2 EGTA, 10 HEPES, 20 phosphocreatine-tris, 2 ATP-Na_2_, 0.2 mM GTP-Na_2_ and 0.1 leupeptin, pH 7.2 with KOH. Pipette resistance was between 3 and 4MΩ. Cell capacitance and series resistance errors were carefully compensated (∼85%) throughout the experiment. The remaining linear capacity and leakage currents were eliminated online using a P/4 subtraction paradigm. Total voltage-gated currents were elicited by applying 66 ms-long depolarizing voltage steps from −70 to +60 mV (10 mV increments), from a holding potential of −70 mV. Sodium and potassium current densities were obtained by dividing the recorded currents for the cell capacitance, measured by integrating the capacitative current evoked by a 10 mV hyperpolarizing voltage step from the holding potential. Discharges of action potentials (APs) were evoked by the injection of 2.5 s-long depolarizing current pulses of increasing amplitude from the resting potential maintained at −70 mV.

### 4.11. Statistical Analyses

For morphological characterization statistics was performed using GraphPad Prism 7 Software and expressed as mean ±S.E.M. *n* > 50 neuronal nuclei for every sample was analyzed. About 50 cells were scored for branching and end points parameters. Kruskal–Wallis non-parametric test was used to make multiple comparisons.

Non-parametric Mann–Whitney test was applied to compare the viability of untreated and treated cells from both control and patient neuronal cultures.

For electrophysiological experiments, mean values were compared with One-way ANOVA and Scheffe’s post-hoc test (normality was tested with the Kolmogorov–Smirnov test and homogeneity of variance with the Levene’s test), and contingency tables were analyzed with the Fisher exact test and Bonferroni correction for multiple comparisons.

## Data Availability

Data on the iPSC lines generated from *CREBBP*-mutated patients P149 and P34 investigated in the present work have been uploaded on the Human Pluripotent European Stem cell Registry: https://hpsreg.eu (accessed on 23 May 2021) under the names IAIi004-A and IAIi002-A, respectively. Data on transcriptome analysis of a set of RSTS patients including P149 and P34 investigated in the present work can be found at https://www.ncbi.nlm.nih.gov/geo/query/acc.cgi?acc=GSE135287 (accessed on 23 May 2021).

## Supplementary Materials

The following are available online at https://www.mdpi.com/article/10.3390/ijms22115777/s1. Figure S1: Workflow of TSA short- and long-term treatment and viability assay. Neural progenitors obtained after initial (35 days) differentiation of iPSCs are thawed and set in culture medium supplemented after three days of cell recovery with TSA 20 nM for 7 days till the stage of 45 days neurons (A) and TSA 0.2 nM for 6 weeks till to mature (>75 days) neurons (B). Young neurons are suitable for morphological analysis and post-mitotic neurons can be used for electrophysiological recordings. Both TSA acute (C) and chronic (D) treatment do not affect cell viability of CTRL and patient neuronal cultures (nt = not significant). Non-parametric Mann–Whitney test was applied. Figure S2: Stepwise procedure to acquire 1024 × 1024 pixel images for morphological analysis of differentiating neurons (40x objective). (A) After setting the measuring scale in micron, each image is split into the three single fluorescence acquisition channels (blue: DAPI; green: TUJ1; red: MAP2) and then binarized and converted into a skeleton. A “Results” window is thereafter displayed, showing for each skeleton in the image various morphological parameters. Here, shown are the selected ones, i.e., the number of end-points, the average length of branches, and the longest shortest path (the shortest joining the two most distant vertices, which corresponds to an average cell length measurement, representing “the neuronal length”). (B) Nuclei analysis is performed on blue single channel binarized images, using the “Analyzed Particles” function: results are shown in a summary table. Figure S3: Analysis with Image J plugin of the length of 45 days neurons from untreated and TSA-treated controls and *CREBBP*-mutated patients. The graph shows that the neuronal length of P34 2 line is significantly reduced (** *p* = 0.0014) as compared to CTRLs and P149 lines and is significantly corrected (#### < 0.0001) after TSA treatment. ns = not significant (Kruskal–Wallis test). Figure S4: Percentages of Multipolar and Uni-Bipolar neurons (45 days) from CTRL and patients lines untreated and TSA or VPA treated. (A,B) Specular distributions of multipolar and uni-bipolar neurons in the indicated controls and patients lines without and with TSA 20 nM for one week. P149 7 and P149 6 lines have significantly less multipolar neurons with respect to CTRLs (** *p* = 0.0098, **** *p* < 0.0001, respectively), while no significant difference is quantified for P34 2 line (ns = not significant). TSA treatment does not impact the neuron layout. (C,D) Specular distributions of multipolar and uni-bipolar neurons in CTRL and P149 7 lines without and with VPA 1 mM for 72 h. A significant effect of VPA in enhancing multipolar neurons percentage is recorded on P149 7 (# *p* = 0.0319) (Kruskal–Wallis non-parametric test). Figure S5: Immunofluorescence characterization of post-mitotic cortical neurons markers. CUX-1 (red), synaptophysin (SYP) (red), glutamatergic subtype specific marker vesicular neurotransmitter transporter (v-GLUT) (green), inhibitory subtype specific marker glutamic acid decarboxylase 65/67 (GAD65/67), (red), and astrocytic marker GFAP (green) in >75 days CTRLs and P149 neurons. Immunostaining of the pan neuronal markers TUJ1 (green) and MAP2 (red) is performed in combination as indicated. Nuclei are stained with DAPI. Scale bars: 100 μm (20×), 25 μm (40× zoomed 4×).
