# Supplementary figures and images for: Histone Deacetylase Inhibitors Ameliorate Morphological Defects and Hypoexcitability of iPSC-Neurons from Rubinstein-Taybi Patients
Source: Int J Mol Sci. 2021 May 28;22(11):5777. doi: 10.3390/ijms22115777 (PMC8197986; doi:10.3390/ijms22115777)

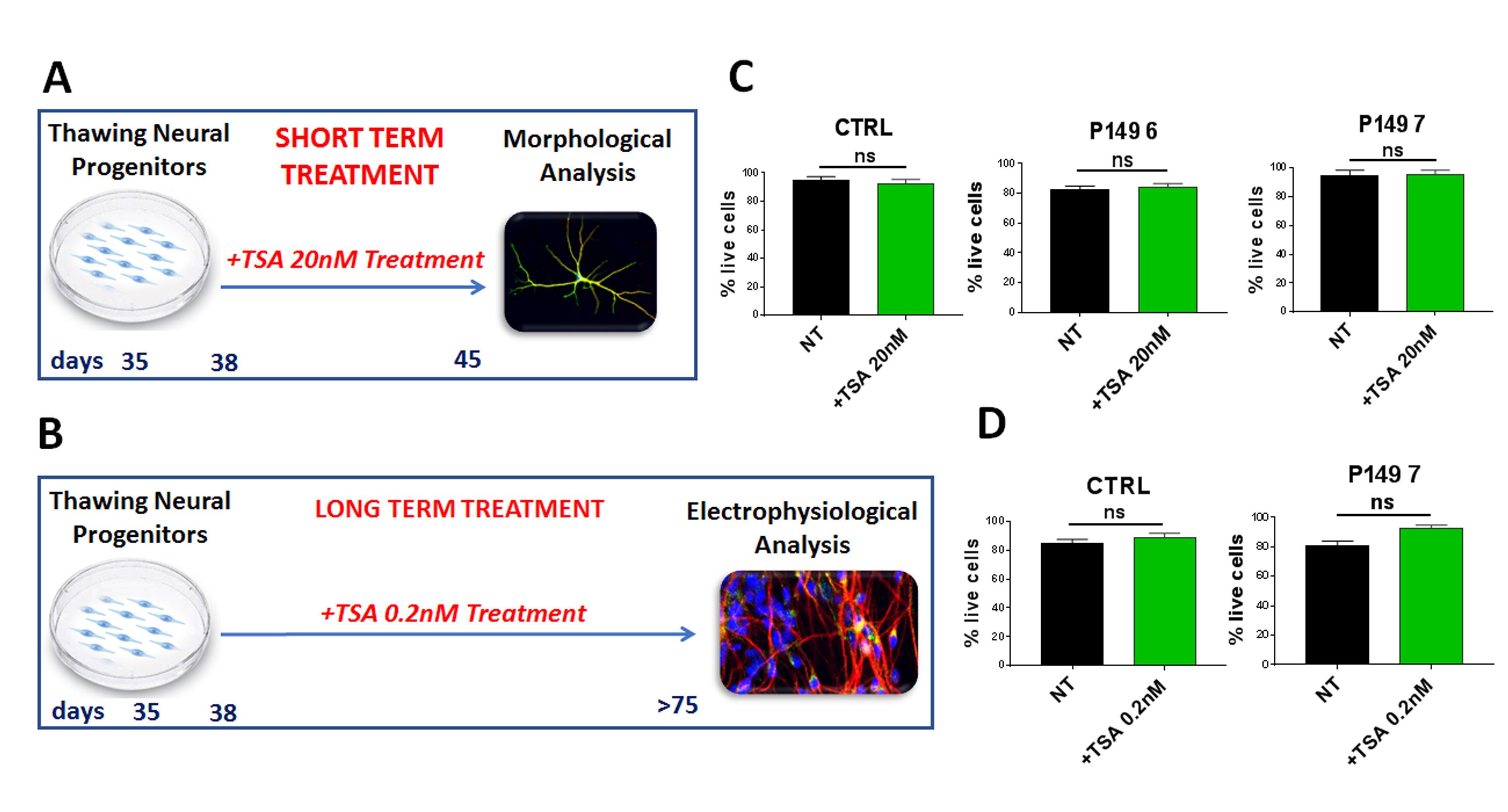

Supplement: Supplementary file 1 [file ijms-22-05777-s001.zip › Suppl 1.tif]

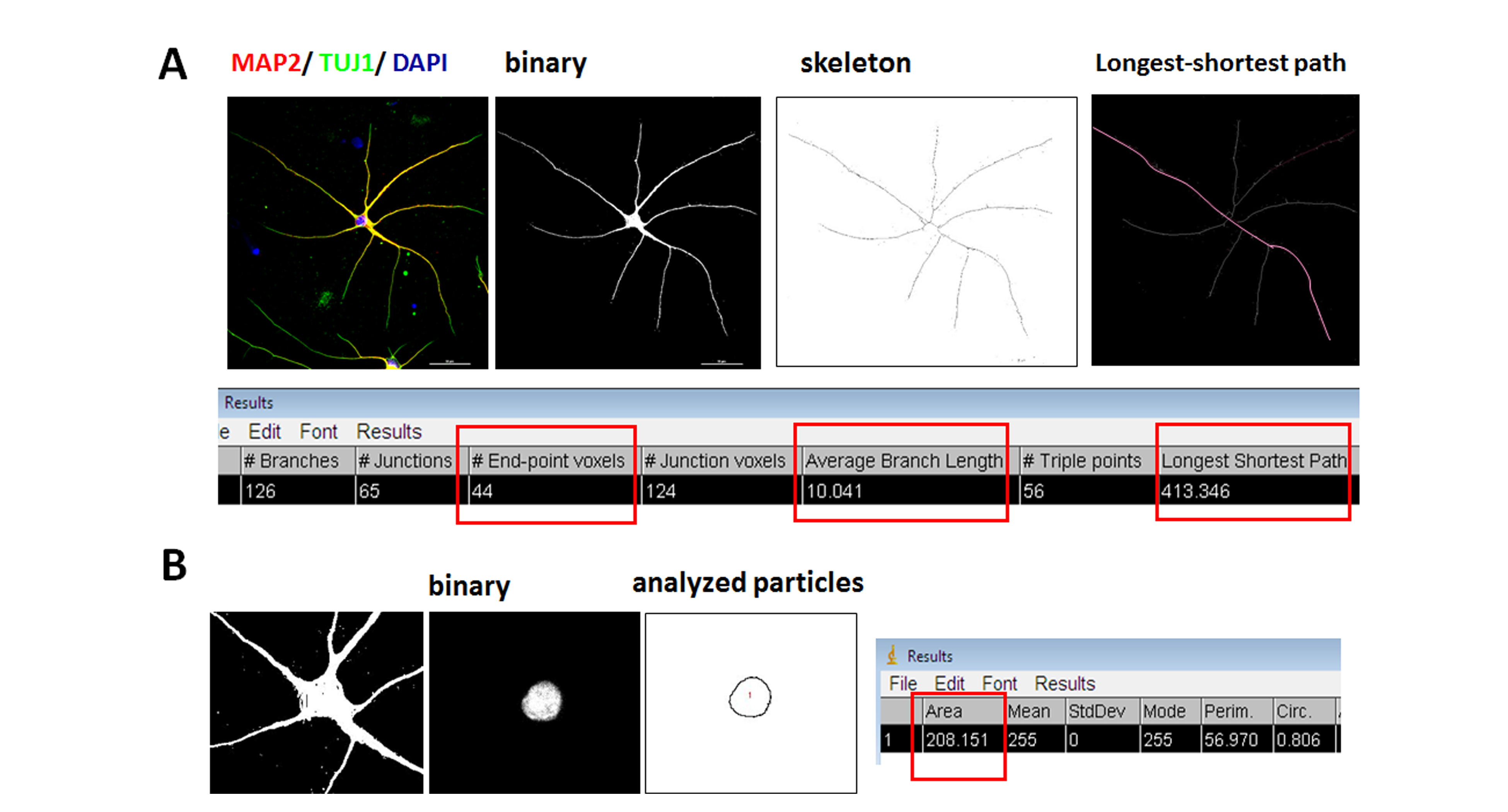

Supplement: Supplementary file 1 [file ijms-22-05777-s001.zip › Suppl 2.tif]

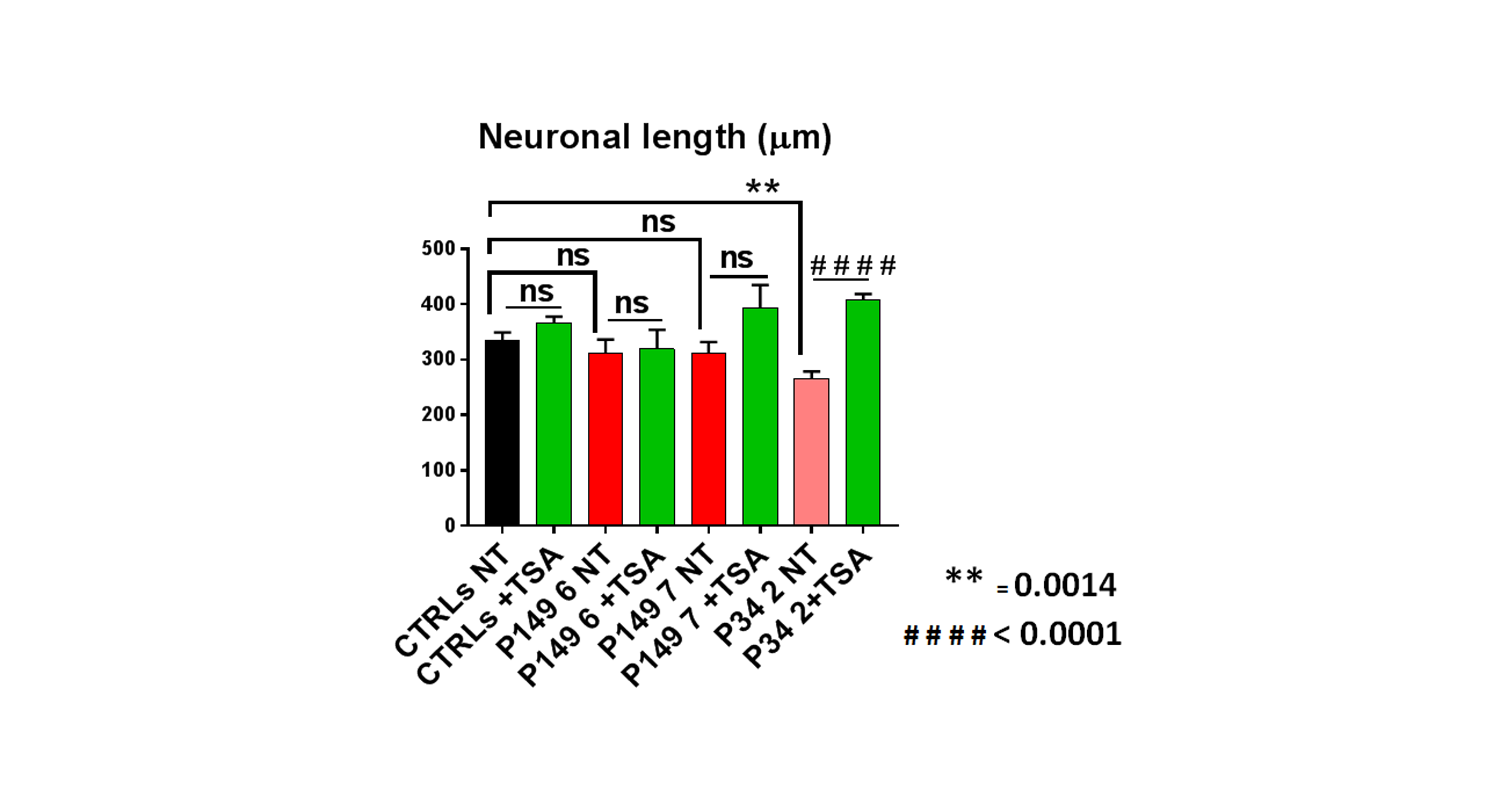

Supplement: Supplementary file 1 [file ijms-22-05777-s001.zip › Suppl 3.tif]

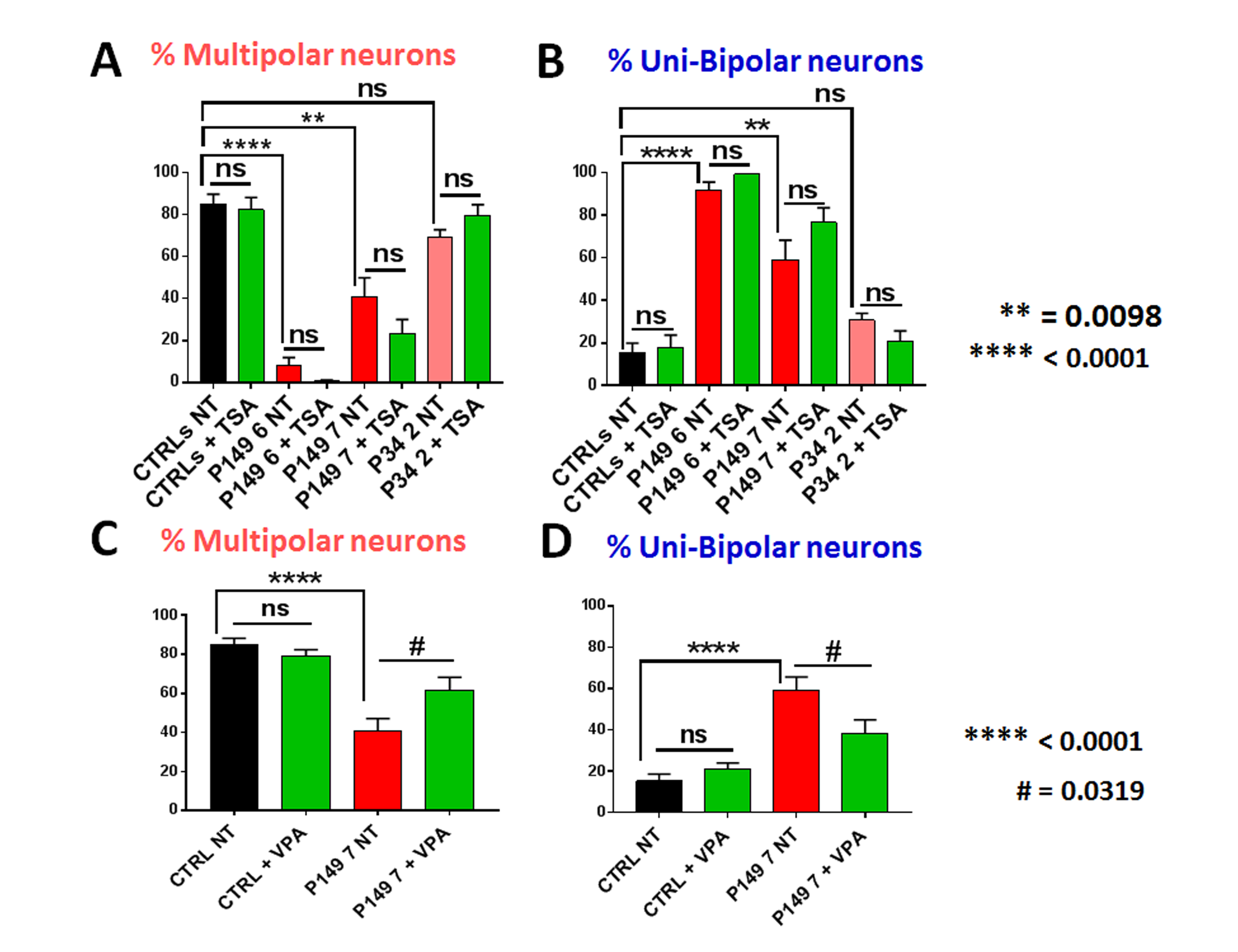

Supplement: Supplementary file 1 [file ijms-22-05777-s001.zip › Suppl 4.tif]

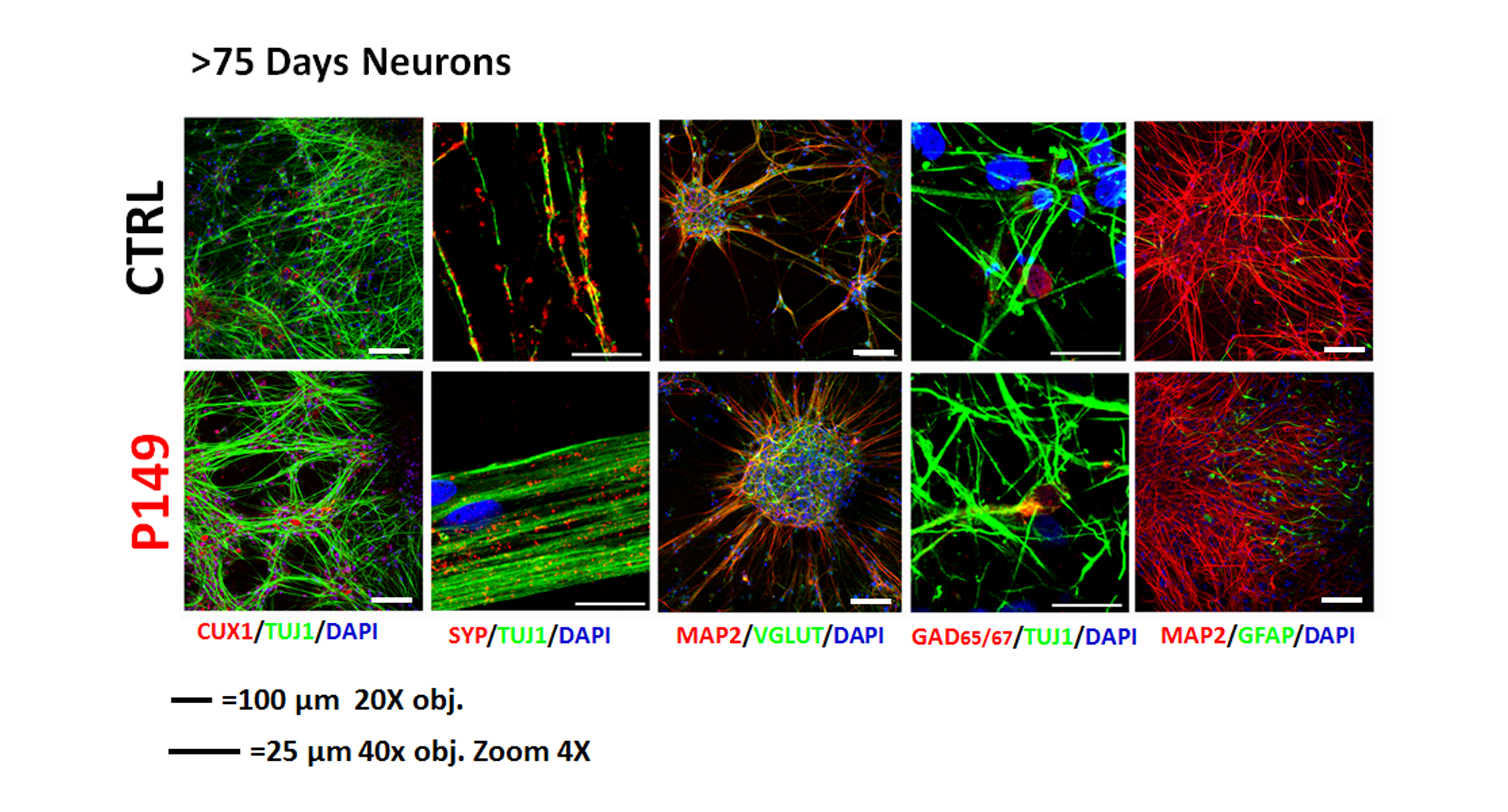

Supplement: Supplementary file 1 [file ijms-22-05777-s001.zip › Suppl 5.tif]

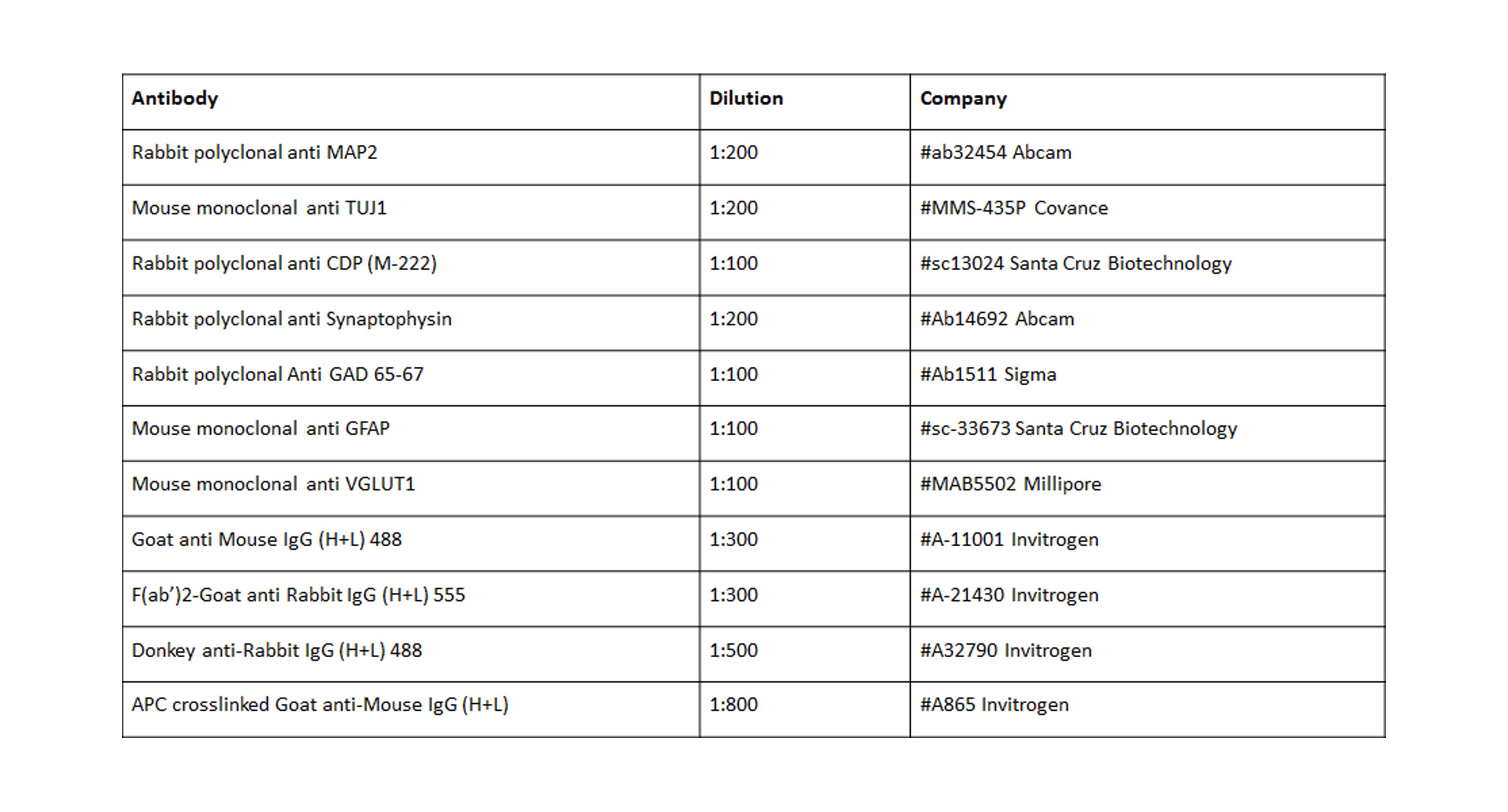

Supplement: Supplementary file 1 [file ijms-22-05777-s001.zip › table 1.tif]
